# Re-exposition to ipilimumab plus nivolumab in metastatic Merkel cell carcinoma

**DOI:** 10.3389/fimmu.2024.1495004

**Published:** 2024-12-05

**Authors:** Valerie Glutsch, Patrick Schummer, Matthias Goebeler, Anja Gesierich, Bastian Schilling

**Affiliations:** ^1^ Department of Dermatology, Venereology and Allergology, University Hospital Würzburg, Würzburg, Germany; ^2^ Department of Dermatology, University Hospital, Goethe University Frankfurt, Frankfurt, Germany

**Keywords:** Merkel cell carcinoma, immunotherapy, resistance, relapse, ipilimumab, nivolumab

## Abstract

**Background:**

Merkel cell carcinoma (MCC) is a rare but highly aggressive cutaneous malignancy. Immune checkpoint inhibition (ICI) with PD-(L)1 blockade has significantly improved treatment outcomes in metastatic disease. In patients with primary resistance to PD-(L)1 inhibition, a high overall response rate (ORR) of 50% to later-line ipilimumab plus nivolumab (IPI/NIVO) has been demonstrated. However, clinical data on patients with progression after an initial response to IPI/NIVO are still lacking.

**Methods:**

Clinical data of three metastatic MCC patients who were re-exposed to IPI/NIVO after progression were retrospectively evaluated.

**Results:**

Two of the three patients showed primary resistance to avelumab with progressive disease, while one patient showed complete response (according to RECIST V.1.1). All three patients received combined ICI with IPI/NIVO as subsequent therapy, resulting in an ORR of ∼ 67%. However, all three patients progressed during follow-up and were re-exposed to IPI/NIVO. With a follow-up period ranging from 6.5 to 37.1 months, no PFS event has been detected. ORR for IPI/NIVO re-exposition was equal to that of initial IPI/NIVO treatment.

**Conclusion:**

In this retrospective follow-up analysis, we observed a response rate of 67% and long-lasting responses after re-exposition to combined ICI in metastatic MCC patients with progression after initial response or disease control upon their first IPI/NIVO treatment. An important observation from this small analysis is that primary resistance to PD-L1 inhibition may result in a better response to IPI/NIVO.

## Introduction

Merkel cell carcinoma (MCC) is a rare but aggressive non-melanoma skin cancer that primarily affects elderly patients (1). For unresectable or metastatic disease, immune checkpoint inhibition (ICI) with the programmed cell death protein 1 (PD-1) inhibitor pembrolizumab or the programmed cell death 1 ligand 1 (PD-L1) inhibitor avelumab have replaced chemotherapy as first-line systemic therapy (2–4). Despite high response rates ranging from 56% to 62%, a significant subset of patients exhibits either primary or acquired resistance to PD-(L)1 blockade (4). For patients with primary resistance to PD-L1 inhibition with avelumab, we recently reported a high overall response rate (ORR) of 50% to combined ICI with ipilimumab plus nivolumab (IPI/NIVO) as subsequent later-line therapy in a multicenter study of the prospective skin cancer registry ADOREG (5). However, median progression-free survival (PFS) was 5.1 months, with 1-year and 2-year PFS landmarks of 42.9% and 26.8%, respectively. These results reflect the urgent clinical need for further subsequent therapy options in case of disease progression or relapse under or after combined ICI with IPI/NIVO. In this retrospective analysis, we report three patients who relapsed or progressed after combined ICI and subsequently were re-exposed to IPI/NIVO.

## Patients and methods

Clinical data of three patients with metastatic MCC who progressed during PD-L1 inhibition with avelumab and were later on treated with IPI/NIVO were retrospectively collected. Data were obtained from electronic medical records by chart review. Due to the retrospective nature of the study and the collection of anonymous patient data, informed consent was waived by the Ethics Committee of the University of Würzburg. Two patients had been reported previously and were included with additional follow-up (5, 6). Progression-free survival (PFS) was calculated from the first course of ICI until tumor assessment which showed progressive disease (PD) toward avelumab (PFS1) or the 1^st^ course of IPI/NIVO treatment (PFS2). PFS and overall survival (OS) for IPI/NIVO re-exposition were calculated from the first course of the IPI/NIVO re-exposition to the last tumor assessment or the last consultation (PFS3 and OS).

## Results

### Patient demographics

Three male patients with metastatic MCC, stage III-IV (UICC 2017), were included in our analysis. The age at first MCC diagnosis ranged from 57 to 72 years. None of the patients were immunosuppressed, either due to a pre-existing hemato-oncological disease or medication. Patient demographics and outcome are summarized in **Table 1**.

**Table 1:** Patient characteristics and course of treatment.

Patients	#1	#2	#3
**Age (years)** at first MCC diagnosis	57	67	72
**Sex**	male	male	male
**Stage** (UICC 2017)	IV	IV	IIIA
**Localization of primary tumor**	gluteal	thigh	unknown
**Avelumab BOR** RECIST 1.1 **Organs involved** (metastases)*	PD LYM	PD LYM	CR LYM
1^st^ IPI/NIVO			
**Organs involved** (metastases)*	peritoneal, perihepatic, interenteric	SKI	LYM
**LDH**	elevated	elevated	normal
**ECOG PS**	0	1	0
**Dosing**	IPI1/NIVO3	IPI1/NIVO3	IPI1/NIVO3
**No. of courses**	4	4	4
**BOR** RECIST 1.1	CR	PR	SD
**Maintenance therapy**	no	no	yes
**PFS after 1^st^ IPI/NIVO** (months)	12.4	22.1	18.9
2^nd^ IPI/NIVO			
**Organs involved** (metastases)*	adrenal	LYM	LYM
**LDH**	elevated	elevated	normal
**ECOG PS**	0	0	0
**Dosing**	IPI1/NIVO3	IPI1/NIVO3	IPI1/NIVO3
**No. of courses**	4	2	4
**BOR** RECIST 1.1	CR	PR	SD
**Maintenance therapy**	yes	yes	no
**PFS after 2^nd^ IPI/NIVO**	no PFS event	no PFS event	no PFS event
**OS/Follow-up** (months)	37.1	21.9	6.5

*at treatment start.

MCC, Merkel cell carcinoma; UICC, Union international contre le cancer; BOR, best overall response; LDH, Lactate dehydrogenase; ECOG PS, Eastern Cooperative Oncology Group Performance Status; PFS, progression free survival; PD, progressive disease; SD, stable disease; PR, partial remission; CR, complete remission; IPI1/NIVO3, Ipilimumab 1 mg/kg + Nivolumab 3 mg/kg; OS, overall survival; LYM, lymphonodal; SKI, skin.

### Pre-therapies

As first-line treatment for metastatic disease, all three patients received the PD-L1 inhibitor avelumab (10 mg per kilogram body weight, mg/kg). The number of courses ranged from two to 21. Two patients showed PD, while one patient showed a complete response (CR) according to RECIST V.1.1 in the first tumor assessment after therapy initiation. The patient who showed complete response to avelumab progressed after 13.4 months while still being on treatment. Treatment-related immune-related adverse events (irAE) of grade II and III (according to Common Toxicity Criteria of Adverse Events, CTCAE 4.03) were observed in only one of three patients. The patient developed pneumonitis grade II and hepatitis grade III after two courses of avelumab and was treated with methylprednisolone.

One patient underwent surgery and radiotherapy after having progressed under avelumab. Another patient received chemotherapy with carboplatin plus etoposide, but showed PD in the first tumor assessment (RECIST V.1.1.) after therapy initiation in between avelumab and IPI/NIVO treatment. The remaining patient received neither systemic therapy nor locoregional treatment in the interim.

All three patients received IPI/NIVO (flipped dosing IPI 1 mg/kg plus NIVO 3 mg/kg) as subsequent later-line therapy. Lactate dehydrogenase (LDH) was elevated in two of three patients. Eastern Cooperative Oncology Group Perfomance Status (ECOG PS) ranged from 0-1. All three patients received four courses of IPI/NIVO. Two out of three patients (1 CR; 1 partial response (PR)) responded to combined IPI/NIVO according to RECIST V.1.1 resulting in an ORR of ∼ 67%. The 3^rd^ patient showed stable disease (SD) and, unlike the two responders, received maintenance therapy with nivolumab 480 mg q4w. No irAE were detected during the 1^st^ IPI/NIVO treatment. PFS2 was 12.2, 22.1 and 18.9 months, respectively.

Of note, the patient who achieved CR after combined IPI/NIVO relapsed early and was initially treated with surgery (PFS 12.2 months).

### Ipilimumab plus nivolumab re-exposition

After relapse or progression, all three patients were re-exposed to combined ICI with IPI/NIVO (flipped dosing IPI 1 mg/kg plus NIVO 3 mg/kg). LDH was elevated in two out of three patients and ECOG PS was 0. Two patients received four courses of IPI/NIVO, while one patient received only two courses due to immune-related hepatitis grade III and immune-related pneumonitis grade II. ORR was consistent to the 1^st^ IPI/NIVO treatment with ∼ 67% (1 CR, 1 PR, 1 SD) according to RECIST V.1.1. The two responders received maintenance therapy with nivolumab. In the patient with SD, we opted against maintenance therapy and instead chose radiotherapy for his stable lymph node metastases.

### Follow-up

After the IPI/NIVO re-exposition, no PFS event has been detected so far. Follow-up/OS was 37.1, 21.9 and 6.5 months, respectively (data cut-off 30.06.2024).

**Figure 1:**
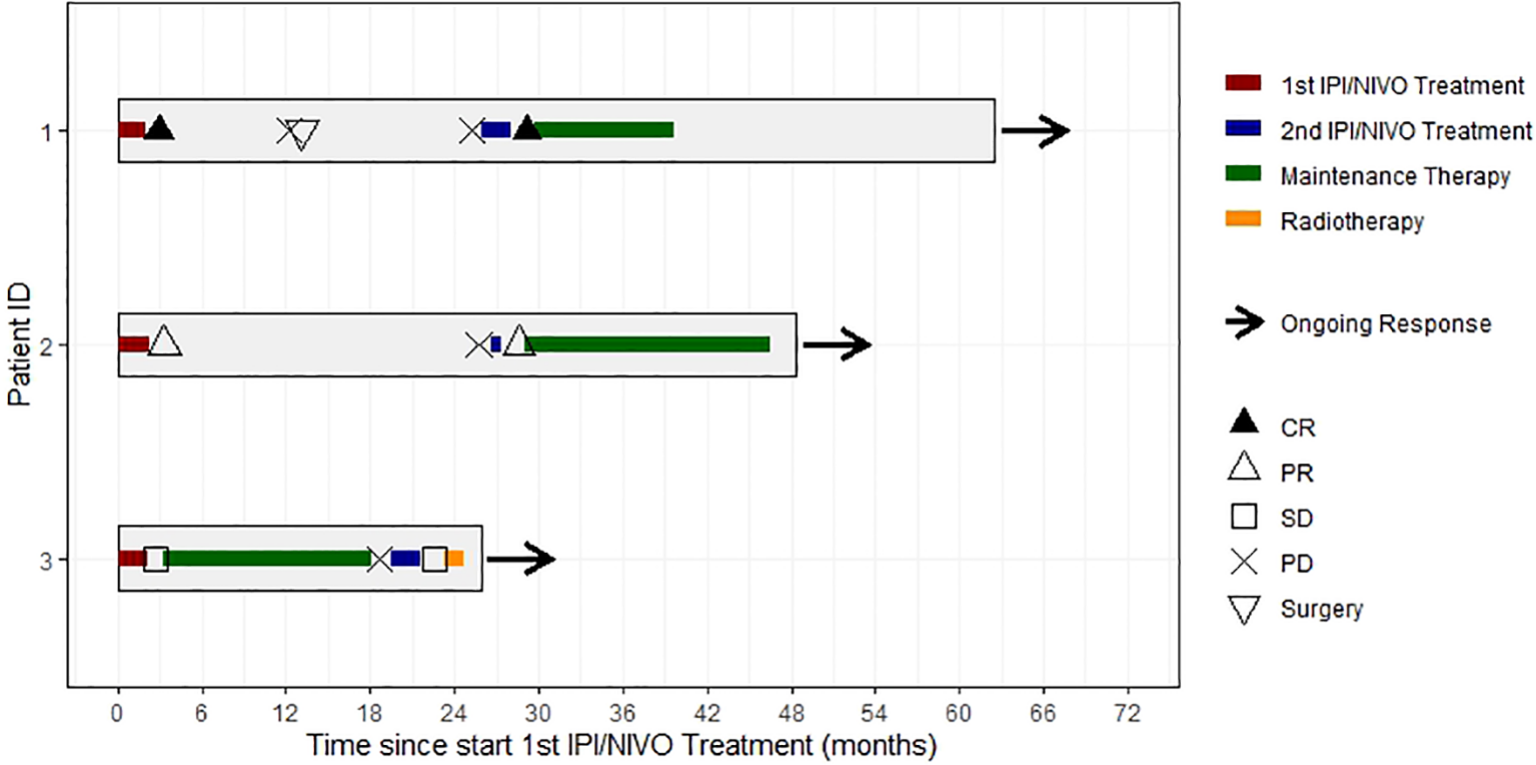
Swimmer’s plot showing individual courses of treatment, response and follow-up of all 3 patients. CR, complete response; PR, partial response; SD, stable disease. PD, progressive disease.

The course of treatment is shown in **Figure 1** for all three patients (**Figure 1**).

## Discussion

In this single-center analysis of three metastatic MCC patients who were re-exposed to combined ICI with IPI/NIVO, we observed a renewed therapy response in two out of three patients. In this small cohort, the ORR to the 2^nd^ IPI/NIVO treatment was equal to the 1^st^ IPI/NIVO treatment. In Europe, PD-L1 inhibition with avelumab is the only approved systemic treatment for locally advanced or metastatic disease. Unfortunately, data on subsequent therapies in case of disease progression or relapse have this far been reported in rather heterogenous and small patient cohorts (7, 8). In a retrospective multicenter analysis of anti-PD-1 refractory melanoma patients, ORR to combined ICI with IPI/NIVO was only 21%, with a one-year overall survival rate of 55% (9). We recently reported a high ORR of 50% with durable responses in a multicenter analysis of 14 metastatic MCC patients with primary resistance to the PD-L1 inhibitor avelumab who were subsequently treated with combined ICI with IPI/NIVO. In this context, primary (not acquired) resistance to first-line PD-L1 (not PD-1) inhibition seems to be crucial for successful subsequent IPI/NIVO treatment since LoPiccolo et al. and Shalhout et al. reported little to no benefit to later-line combined immunotherapy with IPI/NIVO in rather heterogenous cohorts of PD-1/PD-L1 refractory MCC patients (7, 8). Although median OS was not reached, 63.3% of the patients remained alive at the 3-year landmark; median PFS was only 5.1 months, with a relapse in more than 50% of the treated patients at the 1-year landmark (5). Interestingly, 4/7 reported patients (2/4 with maintenance therapy) who primarily responded to IPI/NIVO showed PD or relapsed during follow-up. These data indicate an urgent clinical need not only for subsequent therapies after PD-L1/PD-1 failure but also in later line settings with relapse or PD under or after combined ICI with IPI/NIVO. In our current analysis, PFS after the 1^st^ IPI/NIVO treatment ranged from 12.4 months to 22.1 months, while only one out of three patients received maintenance therapy with nivolumab. Based on the analysis of this small cohort and the literature, a re-challenge with IPI/NIVO appears particularly promising in patients with primary resistance to PD-L1 inhibition and primary deep PR or CR to IPI/NIVO. It remains uncertain if a maintenance therapy with nivolumab could have prevented relapse or disease progression in our two responders after the 1^st^ IPI/NIVO treatment.

Despite the convincing efficacy of later-line IPI/NIVO in metastatic MCC patients who showed primary resistance to PD-L1 inhibition, the recently reported ORR of 100% to 1^st^ line IPI/NIVO in patients with advanced MCC by Kim et al. substantiates considerations to prefer combined ICI with IPI/NIVO to PD-L1 respectively PD-1 monotherapy in the first-line setting (10). However, combined ICI with IPI/NIVO is associated with a high rate of severe irAE (11). In our analysis, IPI/NIVO was surprisingly well tolerated, with only one out of three patients experiencing irAEs of the grades II and III. Interestingly, this patient has tolerated the 1^st^ IPI/NIVO treatment without any irAE and showed the same irAE during re-challenge with IPI/NIVO as during first-line PD-L1 inhibition with avelumab. Notably, all three patients received IPI 1 mg/kg plus NIVO 3 mg/kg (“flipped dose”, chosen according to the CheckMate-358 study, NCT02488759). Since the toxicity and rate of severe irAE of combined ICI seems to depend mainly on the dosing of IPI, using the “flipped dose” in this cohort might explain the low rate of severe irAE at least in part (12).

Our analysis has some limitations. The main limitation is the very small number of patients, as well as the retrospective data collection. Based on the data of three patients, a formal analysis of PFS and OS is not reasonable. Therefore, the course of treatment of our patients was alternatively presented as descriptive analysis (**Figure 1**).

In conclusion, this retrospective follow-up analysis of metastatic MCC patients who relapsed or progressed during or after later-line combined ICI with IPI/NIVO showed a renewed response, with durable responses to re-exposition with IPI/NIVO, primarily in former IPI/NIVO responders. An important observation from this small analysis is that primary resistance to PD-L1 inhibition with avelumab is likely to result in a better response to IPI/NIVO. To confirm this observation, a prospective randomized trial on the efficacy of IPI/NIVO, possibly also as 1^st^ line therapy in this entity, is desirable.

## Data Availability

The original contributions presented in the study are included in the article/supplementary material. Further inquiries can be directed to the corresponding author.
